# Optimization of CMT Characteristic Parameters for Swing Arc Additive Manufacturing of AZ91 Magnesium Alloy Based on Process Stability Analysis

**DOI:** 10.3390/ma16083236

**Published:** 2023-04-19

**Authors:** Zhongrui Zhang, Junqi Shen, Shengsun Hu, Yang Chen, Chengxuan Yin, Xianzheng Bu

**Affiliations:** 1 Tianjin Key Laboratory of Advanced Joining Technology, Tianjin University, Tianjin 300354, China; 2 School of Materials Science and Engineering, Tianjin University, Tianjin 300354, China; 3 International Institute for Innovative Design and Intelligent Manufacturing of Tianjin University, Shaoxing 312000, China; 4 Beijing Hangxing Machinery Co., Ltd., Beijing 100013, China

**Keywords:** magnesium alloy, swing arc additive manufacturing, CMT, droplet transfer, stability analysis, parameter optimization

## Abstract

The droplet transfer behavior and stability of the swing arc additive manufacturing process of AZ91 magnesium alloy based on the cold metal transfer (CMT) technique were studied by analyzing the electrical waveforms and high-speed droplet images as well as the forces on the droplet, and the Vilarinho regularity index for short-circuit transfer (*IV_SC_*) based on variation coefficients was used to characterize the stability of the swing arc deposition process. The effect of the CMT characteristic parameters on the process stability was investigated; then, the optimization of the CMT characteristic parameters was realized based on the process stability analysis. The results show that the arc shape changed during the swing arc deposition process; thus, a horizontal component of the arc force was generated, which significantly affected the stability of the droplet transition. The burn phase current I_sc_wait presented a linear function relation with *IV_SC_*, while the other three characteristic parameters, i.e., boost phase current I_boost, boost phase duration t_I_boost and short-circuiting current I_sc2, all had a quadratic correlation with *IV_SC_*. A relation model of the CMT characteristic parameters and *IV_SC_* was established based on the rotatable 3D central composite design; then, the optimization of the CMT characteristic parameters was realized using a multiple-response desirability function approach.

## 1. Introduction

Metal resource consumption is increasing due to the needs of economic development, and most countries have made great efforts to strike a balance between economic growth and environmental protection. This pressure can be effectively reduced using high-performance lightweight metals, so their exploration and use have become popular research topics. As one of the Earth’s most abundant metal elements, magnesium has a density of only 1.74 g/cm^3^. Magnesium alloys have the characteristics of high specific strength and specific stiffness; excellent shock resistance and impact resistance; and good properties of thermal conductivity, damping and electromagnetic shielding. They also have the advantages of easy processing and recycling [1,2,3,4]. As a result, it has been discovered that magnesium alloys have significant application value and a wide range of potential applications in the fields of transportation, national defense, communication and medicine, and other industrial fields [5]. In engineering applications, casting is still the main processing method for magnesium alloys [6]. However, the tendency to obtain coarse grains when casting magnesium alloys results in poor mechanical properties and thus severely constrains the further application of magnesium alloys.

The direct production of large components and complex precision parts is greatly facilitated by additive manufacturing (AM). As one of the important techniques of AM, wire arc additive manufacturing (WAAM) is a direct energy deposition technique that uses arc welding procedures to fabricate components using layer-by-layer deposition, and it has been widely used for manufacturing parts of aluminum alloy [7,8], nickel-based alloy [9,10], stainless steel [11] and other metal materials. However, porosity, grain coarsening and thermal cracking are more likely to occur during the conventional WAAM process of magnesium alloys due to the high sensitivity to heat inputs induced by its unique physical and chemical properties [12]. As an improved technique of gas metal arc welding (GMAW), cold metal transfer (CMT) can realize a low-heat-input process with the precise control of the wire movement and the welding parameters by using a push–pull mechanism coupled with a high-speed digital process control system; thus, it helps to solve the above problems. Moreover, a stable and spatter-free WAAM process for magnesium alloys is made possible by the retraction control of the filler wire during the short-circuiting stage [13]. Therefore, the CMT technique shows significant technical advantages and broad application prospects in the fields of the WAAM of magnesium alloys.

By analyzing the electrical signals during the GMAW process, the arc stability can be determined as an index of the stability of the welding process [14]. Suban and Tusek [15] used the probability distribution of welding current to evaluate the influence of shielding gas components on arc stability during the GMAW process of low-carbon steel and found that the high current intensity in the short-circuit stage led to strong spatter caused by the breakage of the liquid bridge, making the welding process unstable. Chen et al. [16] explored the effect of the CMT characteristic parameters on the welding process stability of mild steel using the probability distributions of the CMT period and short-circuit time and indicated that fairly regular and stable processes could be achieved when the boost duration ranged from 1.6 ms to 3.6 ms at the boost current of 300 A. Singh et al. [17] used cyclograms of welding current vs. welding voltage to characterize arc stability and the spatter during the CMT weld-brazing process of aluminum/steel dissimilar alloys, and the results showed that the good repeatability of the cyclograms represented strong arc stability and the absence of spatter. The optimization of the welding parameters (i.e., welding current and welding voltage) by realizing the accurate control of the different stages of the GMAW process can reduce spatter and improve the stability of the welding process. Zhang et al. [18] achieved the desired one-droplet-per-pulse (ODPP) transfer by selecting the appropriate combination of the duration and amplitude of the peak current in the pulsed GMAW (GMAW-P) process of titanium alloy, and the stability of the GMAW-P process was greatly improved due to the precise control of the heat input in different stages of the droplet transition process. Subsequently, a much stronger droplet oscillation with a significantly lower heat input was realized with the active control of the current waveform during the GMAW-P process [19], which further increased the process stability.

Swing arc welding can effectively improve the weld appearance and grain refinement [20] and can also significantly reduce the formation of defects such as cracks and porosity [21,22]. In addition, the application of swing arc can increase the width of the deposited layer, thus improving the efficiency of WAAM [23]. However, much of the research on swing arc welding/deposition focuses on the microstructure and properties of the welded joint or deposited layer, while there is little analysis on the process stability when the swing arc is introduced. For example, though the spatter issue occurs when the swing arc is introduced [23], it has not attracted the attention of researchers. As is well known, welding spattering can lead to problems such as low wire utilization, poor bead formation, poor mechanical performance of the obtained joints and an extensive post-cleaning process [24,25,26]. In the multi-layer and multi-pass WAAM process, the spatter on the deposited pass is extremely detrimental to the deposition process stability of the subsequent pass and eventually to the performance of the WAAM component. Therefore, in order to ensure the appearance quality and performance of the deposited component, it is necessary to conduct research on the process stability of CMT swing arc deposition and the optimization of the CMT characteristic parameters.

As mentioned above, the introduction of the swing arc can cause spatter issues during the deposition process. Therefore, it is necessary to analyze the effect of the forces on the droplet during the CMT-WAAM process of AZ91 magnesium alloy based on the static equilibrium when the swing arc is applied, which can provide the theoretical analysis of spatter formation during the swing deposition process. Then, the influence of the CMT characteristic parameters in different stages on the stability of the swing arc deposition process can be investigated using the Vilarinho regularity index for short-circuit transfer (*IV_SC_*) based on the coefficient of variation of electrical signals, as well as droplet transfer analysis. Finally, the stable swing arc deposition process can be achieved by optimizing the CMT characteristic parameters based on the above-mentioned process stability analysis.

## 2. Experimental Methodology

The substrate used in the experiment was an as-cast AZ91 magnesium alloy plate with dimensions of 200 mm × 150 mm × 8 mm, and the deposited material was AZ91 magnesium wire with a diameter of 1.2 mm. The chemical composition of the substrate plate and wire is illustrated in Table 1. The welding system was composed of a Motoman HP6 industrial robot and a Fronius Advanced 4000R welding machine equipped with a RCU 5000i controller, and the CMT program of C1904 was used to conduct the deposition process.

Before the deposition, the surface of the substrate plate was polished and wiped with ethanol to remove the oxide film and oils. The contact tip-to-work distance (CTWD) was maintained at 12 mm, and the welding gun was perpendicular to the substrate. Pure argon was used as the shielding gas, and the flow rate was 15 L/min. Based on the trial experiments and our previous research studies [27,28], the pre-flow time and post-flow time of the shielding gas were set at 1 s and 2.5 s, respectively, to ensure the protection effect on the start and end positions. During the deposition process, the substrate plate was fixed with a clamping device to prevent deformation. The introduction of the swing arc was realized using the ‘single oscillation’ weaving mode of the robot. The swing frequency and the swing amplitude were 5 Hz and 5 mm, respectively, and the dwell time on both sides and in the middle were both 0.1 s. The traveling speed of the welding torch was kept at 0.54 m/min.

**Figure 1:**
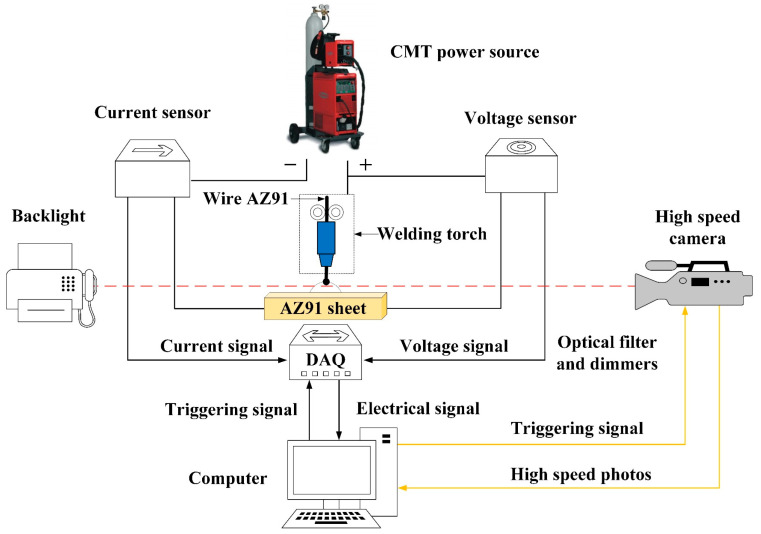
Schematic of the acquisition system.

Figure 1 shows the acquisition system consisting of a high-speed camera and an electrical signal acquisition device. The high-speed camera with an acquisition frequency of 500 fps was used to capture the droplet transfer, and a 1000 W high-voltage short-arc spherical xenon lamp was employed as the backlight source. A Hall current sensor and a Hall voltage sensor were used to capture the welding current and welding voltage signals, respectively, and a PCI-1742 data acquisition card with the sampling frequency of 50 kHz was used to send the electric signals to the computer. The program compiled using LabVIEW software was used to synchronously trigger the high-speed camera and electrical signal acquisition system.

**Figure 2:**
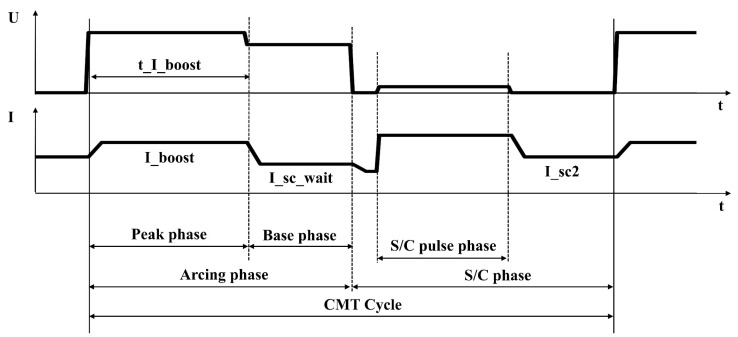
Schematic of voltage and current waveforms during CMT welding process of Mg alloy.

A low-heat-input and spatter-free CMT welding process can be achieved as mentioned above, with the precise control of welding parameters such as welding current using high-speed digital process control. The schematic of the welding voltage and current waveforms used in the CMT welding of magnesium alloy is shown in Figure 2. There are four main characteristic parameters that can be controlled during the CMT welding process, i.e., boost phase current I_boost (A), boost phase duration t_I_boost (ms), burn phase current I_sc_wait (A), and short-circuiting current I_sc2 (A). When the arc is ignited after the detachment between the wire and the molten pool, the welding current is rapidly changed from I_sc2 to I_boost so as to keep the arc burning and the wire melting, and the duration of I_boost (i.e., the current of the peak phase) is controlled by t_I_boost. Then, the welding current is reduced to I_sc_wait, and the droplet gradually moves closer to the molten pool during the base phase. The short-circuit phase (S/C phase) occurs when the droplet comes into contact with the molten pool, and the welding current is immediately adjusted to the S/C pulse current and then is decreased to I_sc2. Finally, the droplet detaches from the wire into the molten pool, and a new CMT cycle occurs.

The precise control of the process stability in different stages can be achieved by adjusting the above CMT characteristic parameters. Based on the default electrical waveforms of the C1904 program at the wire feeding speed of 9 m/min (i.e., electric waveforms of sample number 1 in Table 2 as the basis for adjustment), the process stability under the conditions of different CMT characteristic parameters was analyzed. The CMT characteristic parameters for the swing arc deposition of Mg alloy are listed in Table 2.

## 3. Results and Discussion

### 3.1. Cause Analysis of Spatter under Swing Arc

The forces on the droplet can be divided into two categories according to their effects on the droplet transition to the molten pool: positive ones and negative ones. The main forces acting on the droplet during the CMT deposition process include gravity (*G*), surface tension (*F*_γ_), arc force (*F*_arc_) and mechanical retraction force (*F*_b_) [29]. The reason for the formation of spatter during the CMT swing arc deposition process of Mg alloy can be investigated by analyzing the forces on the droplet. Among the above forces, *G* is determined by the droplet quality (i.e., the amount of melted wire); *F*_γ_ is determined by the surface tension coefficient and the curvature radius of the liquid surface; *F*_arc_ is related to the current and the arc shape; and *F*_b_ mainly depends on the retraction speed of the wire. The introduction of the swing arc has little effect on the *G*, *F*_γ_ and *F*_b_ of the droplet, but it leads to an obvious change in *F*_arc_ due to the arc shape variation and thus affects the stability of the deposition process. During the CMT swing arc deposition process of Mg alloy, the arc force mainly includes electromagnetic force, plasma fluid force and reaction force of metal vapors.

**Figure 3:**
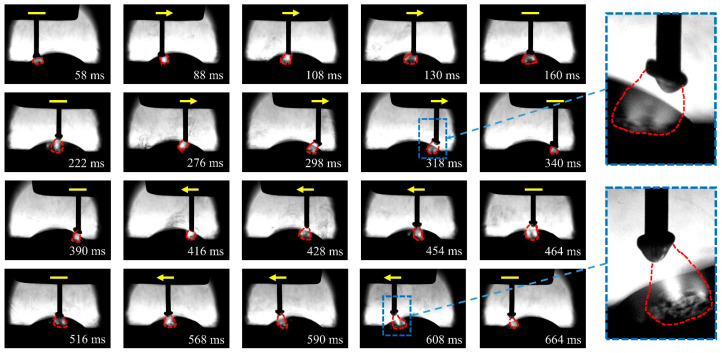
Arc shape variation during the swing deposition process.

Due to inertia, the arc and the droplet tend to keep moving in the same direction when the torch swings. Therefore, the arc shape becomes asymmetrical during the swing deposition process, as shown in Figure 3. The arc force during the swing deposition process is different from that during the linear deposition process, because the arc force is related to the arc shape [30]. Since the arc is bell-shaped and symmetrical along the wire axis during the linear deposition process, the arc force is also symmetrical, and there is no component in the horizontal direction. However, in the swing deposition process, the torch movement alters the shape of the arc and reduces its straightness, resulting in a horizontal component of the arc force acting on the droplet.

**Figure 4:**
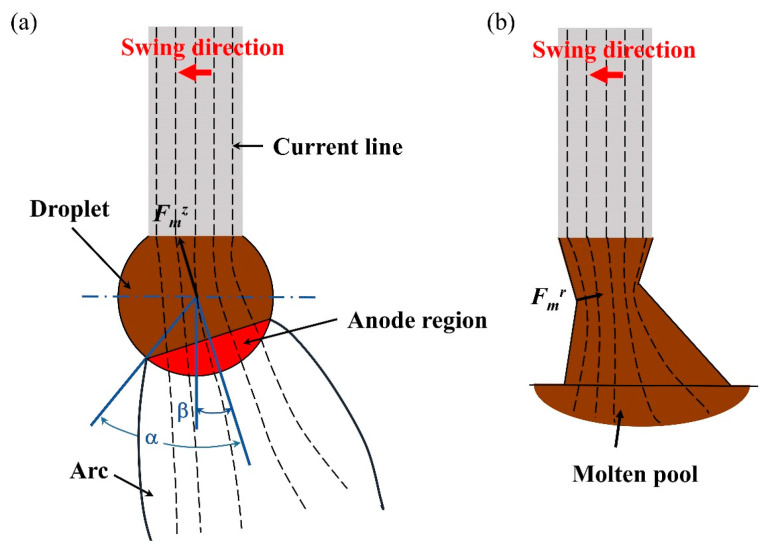
The electromagnetic force on the droplet: (**a**) formative stage; (**b**) transitional stage.

The electromagnetic force (*F_m_*) is a vector with magnitude and direction, and it can be determined using Equation (1):

(1) $$F_{m}=J\;\times \;B$$

where *J* is the current density and *B* is the electromagnetic flux density. During the linear deposition process, the current density is uniform, so the axial electromagnetic force is directed along the wire. However, during the swing arc deposition process, the offset arc acts on the side of the droplet, resulting in the uneven distribution of current density, as shown in Figure 4a. Therefore, the axial electromagnetic force hinders the droplet transition when the droplet diameter is greater than that of the wire. The axial electromagnetic force ($F_{m}{}^{z}$) on the droplet can be calculated according to Equation (2) [31]:

(2) $$F_{m}{}^{z}=\frac{\mu_{0}I_{\mathrm{arcing}}{}^{2}}{4\pi }\left[ln\frac{r}{R}\sin\alpha \;-\frac{1}{4}-\frac{1}{1\;-\cos\alpha }+\frac{2}{{(1\;-\cos\alpha )}^{2}}\ln\frac{2}{1+\cos\alpha }\right]$$

where *μ*_0_ is the dielectric permeability, *I_arcing_* is the current of the CMT arcing phase (i.e., I_boost and I_sc_wait) and *α* is the arc anode half angle.

When the droplet comes into contact with the molten pool, the change in the droplet shape leads to a change in the current density, as shown in Figure 4b. At this moment, a radial electromagnetic force is generated at the necking, at which time the necking shrinks under the action of the radial electromagnetic force, thus promoting the droplet transition to the molten pool. The radial electromagnetic force ($F_{m}{}^{r}$) can be calculated according to Equation (3):

(3) $$F_{m}{}^{r}=\frac{\mu_{0}I_{sc}{}^{2}}{4\pi }\left(R^{2}-{\;r}_{0}{}^{2}\right)$$

where *I_sc_* is the current in the CMT short-circuit phase and *r*_0_ is the radius of the necking.

**Figure 5:**
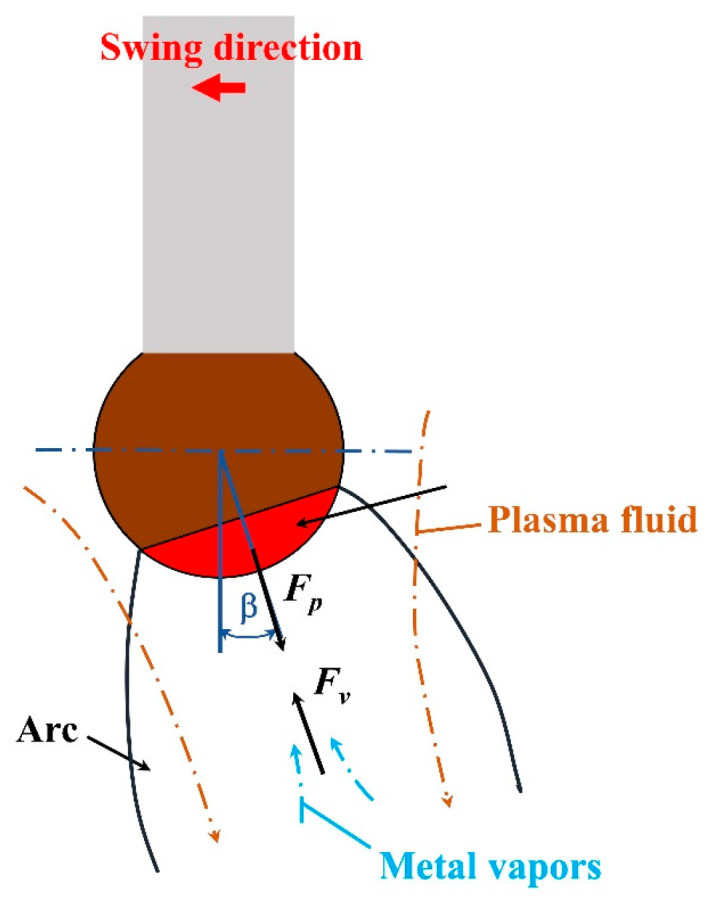
The plasma fluid force and reaction force of metal vapors on the droplet.

The pressure near the electrode is greater than that near the substrate plate due to the uneven electromagnetic force inside the arc, and this pressure difference causes the particles in the arc to move towards the substrate, forming a flow pressure called plasma fluid force. The plasma fluid force (*F_p_*) acting on the droplet can be calculated with Equation (4) [32]:

(4) $$F_{p}{=C}_{D1}A_{p1}(\frac{\rho_{p}v_{p}{}^{2}}{2})$$

where *C_D_*_1_ is the fluid drag coefficient, *A_p_*_1_ is the area of fluid action, *ρ_p_* is the plasma density and *v_p_* is the plasma movement velocity. As shown in Figure 5, the plasma fluid force acts along the arc axis, and the plasma fluid moves in the negative direction of the arc pressure gradient, thus promoting the droplet transition.

During the CMT deposition process, metal vapors are formed due to the low evaporation temperature of the magnesium alloy, and the reaction force of metal vapors pushes the droplet away from the arc axis [5]. The reaction force of the metal vapors (*F_v_*) is also a kind of fluid force, and its magnitude and direction are determined by the flow and direction of the metal vapors. *F_v_* can be calculated according to Equation (5) [32]:

(5) $$F_{v}{=C}_{D2}A_{p2}(\frac{\rho_{v}v_{v}{}^{2}}{2})$$

where *C_D_*_2_ is the metal vapor drag coefficient, *A_p_*_2_ is the area of metal vapor action, *ρ_v_* is the metal vapor density and *v_v_* is the metal vapor movement velocity.

The magnitude and direction of the arc force are related to the welding current and arc shape. During the CMT swing arc deposition process, the magnitude of the arc force does not change because the current remains stable. However, the change in the arc shape caused by the introduction of the swing arc alters the direction of the arc force and generates a component in the horizontal direction, which could lead to large spatter, especially during the multi-layer and multi-pass WAAM process. By adjusting the CMT characteristic parameters, it is achievable to control the forces acting on the droplet in order to eliminate the spatter and guarantee the stable droplet transition process.

### 3.2. Effect of the CMT Characteristic Parameters on Process Stability

**Figure 6:**
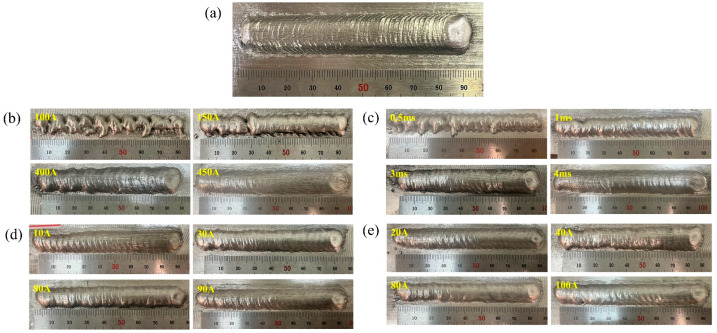
Appearance of the deposited layers: (**a**) Sample number 1; (**b**) Samples number 2–5; (**c**) Samples number 6–9; (**d**) Samples number 10–13; (**e**) Samples number 14–17.

The appearance of the deposited layer is shown in Figure 6. It is obvious that the aforementioned CMT characteristic parameters, i.e., boost phase current I_boost (A), boost phase duration t_I_boost (ms), burn phase current I_sc_wait (A) and short-circuiting current I_sc2 (A), had a significant impact on the appearance of the deposited layer due to their effects on the process stability.

The heat input (*HI*) of the CMT deposition process can be calculated according to Equation (6):

(6) $$HI=\frac{\eta \sum_{i=1}^{n}\mathrm{UiIi}}{{\mathrm{nv}}_{t}}$$

where *n* is the number of electrical signal samples; *U_i_* and *I_i_* are the instantaneous voltage and instantaneous current, respectively; *v_t_* is the traveling speed; and *η* is the thermal efficiency factor, which was taken as 0.7 in this paper. As shown in Table 3, the CMT characteristic parameters of t I_boost and t_I_boost had a significant effect on the heat input, while I_sc_wait and I_sc2 had little influence on the heat input.

One way to characterize the process stability of CMT deposition is to analyze the stability of the electrical signals, and the common indicators for this analysis include the probability density distribution, *U-I* diagram, standard deviation and coefficient of variation. Probability density distribution and *U-I* diagram can provide a visual representation of the stability of the deposition process, but it is difficult to analyze them quantitatively. The coefficient of variation can not only provide the quantitative characterization of process stability for visual comparison but also eliminate the effects of standard deviation induced by the differences in measurement scales and magnitudes. Therefore, the coefficient of variation can provide a more accurate characterization of electrical signal dispersion. In CMT deposition, the process stability can be characterized using the Vilarinho regularity index (*IV_SC_*) related to the coefficient of variation [33,34], which is calculated as shown in Equation (7):

(7) $${IV}_{SC}=\frac{\sigma_{\mathrm{tsc}}}{t_{sc}}+\frac{\sigma_{\mathrm{tarcing}}}{t_{\mathrm{arcing}}}$$

where *σ_tsc_* is the standard deviation of the short-circuit time, *σ_tarcing_* is the standard deviation of the arc burning time, *t_sc_* is the mean short-circuit time and *t_arcing_* is the mean arc burning time. As mentioned, because the regularity index (*IV_SC_*) takes into account not only the standard deviation but also the mean value, a lower *IVsc* indicates a more regular transition (i.e., stable process). Table 3 shows the *IV_SC_* values with different CMT characteristic parameters.

It can be seen that the deposited layer was poorly formed and that the surface had clear ‘grooves’ at I_boost of 100 A (i.e., sample number 2) or t_I_boost of 0.5 ms (i.e., sample number 6), as indicated in Figure 6b,c. The unstable deposition process, as well as low heat input, resulted in a small amount of molten metal, poor wettability of the molten metal and discontinuous deposition process, eventually leading to a very poorly formed deposited layer.

**Figure 7:**
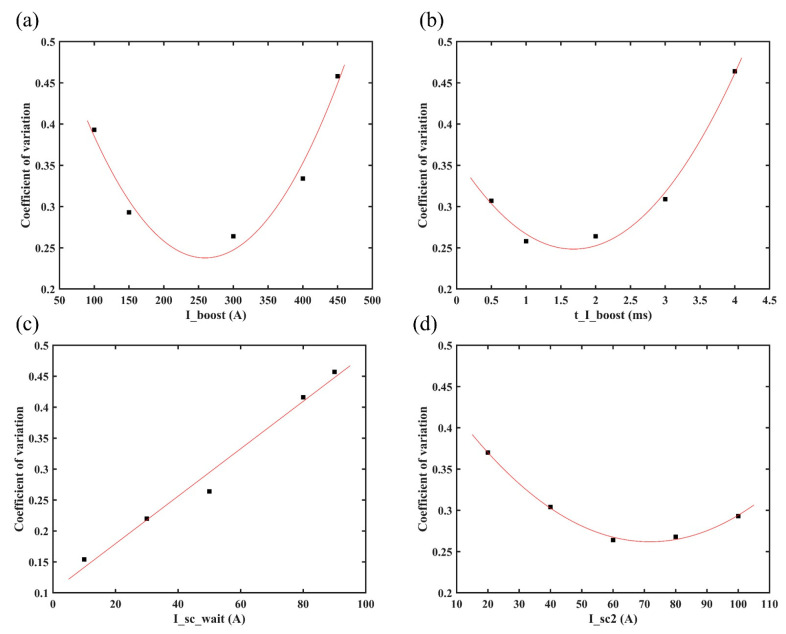
*IV_SC_* of CMT deposition process at different (**a**) I_boost, (**b**) t_I_boost, (**c**) I_sc_wait and (**d**) I_sc2.

The change in electrical signal stability can reflect the trend change of the droplet transition. Figure 7 shows the regression analysis results of the CMT characteristic parameters and coefficients. It is obvious that I_boost, t_I_boost and I_sc2 both displayed a quadratic correlation with *IV_SC_*, while I_sc_wait displayed a good linear function relation with *IV_SC_*.

**Figure 8:**
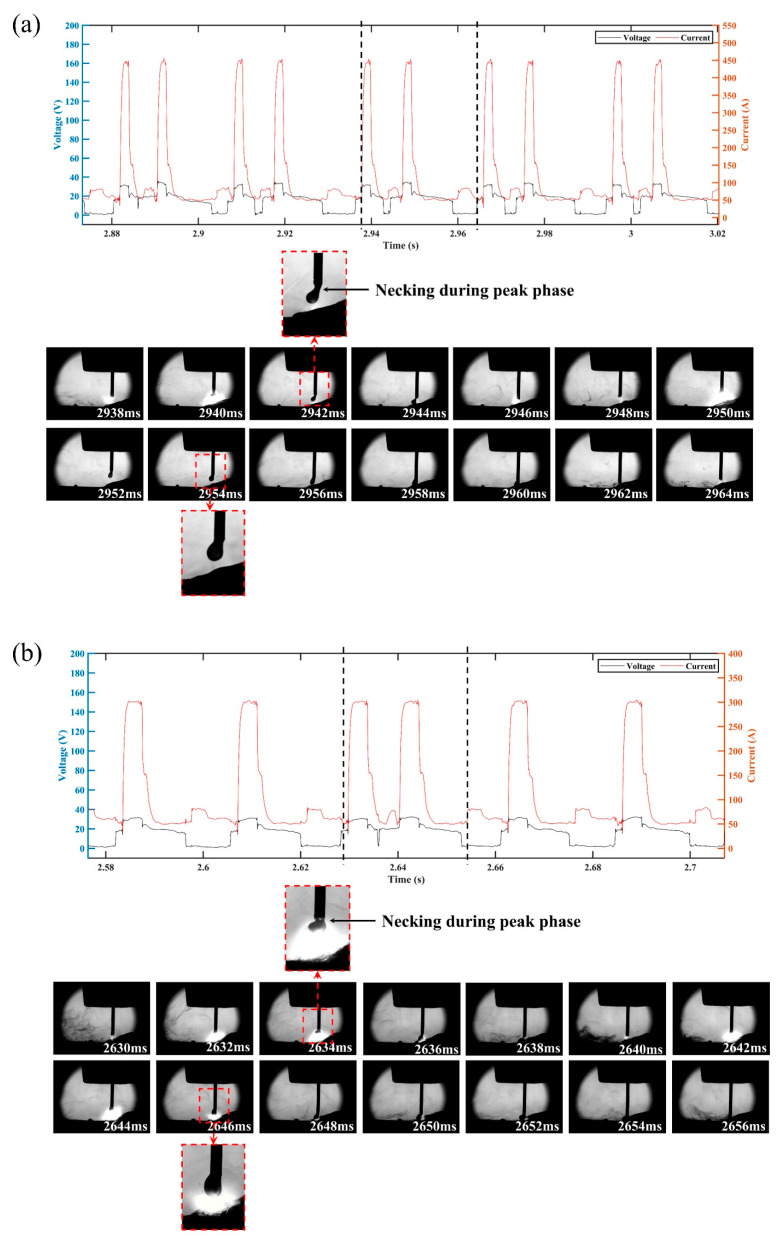
Electrical waveforms and droplet transfer process at (**a**) I_boost = 450 A and (**b**) t_I_boost = 4 ms.

In order to clarify the effects of the CMT characteristic parameters on the droplet transition and process stability, the droplet transition process and the synchronous electrical signal waveforms were analyzed, and the high-speed droplet images corresponds to the area of electrical signal waveforms between the dotted lines. Figure 8 shows the electrical signal waveforms and the corresponding droplet transfer process at I_boost of 450 A (i.e., sample number 5) and t_I_boost of 4 ms (i.e., sample number 9). The electrical signal in both situations showed abnormal CMT cycles with very short duration, which manifested in an extremely short period in the base phase and the short-circuit phase. The corresponding droplet transition process showed that when the abnormal CMT cycle occurred, the droplet directly made contact with the molten pool to form a short-circuit phase and then rapidly detached from the wire. Therefore, the abnormal CMT cycle had no obvious base phase to maintain the arc burning nor short-circuit phase to facilitate the detachment of the droplet, thus leading to the instable deposition process. The high heat input of the peak phase not only melted more metal, but also reduced the viscosity coefficient of the liquid metal [35], so the droplet was prone to necking during the peak phase, and the radial electromagnetic force became a motive force for the droplet transition at that moment. Due to the rapid transfer of the droplet to the molten pool caused by the gravity and electromagnetic forces, the abnormal CMT cycle occurred. As shown in Figure 8a, when I_boost was 450 A, the high peak current led to a great instantaneous electromagnetic pinch force, resulting in a high probability of necking; thus, a greater number of abnormal CMT cycles appeared.

**Figure 9:**
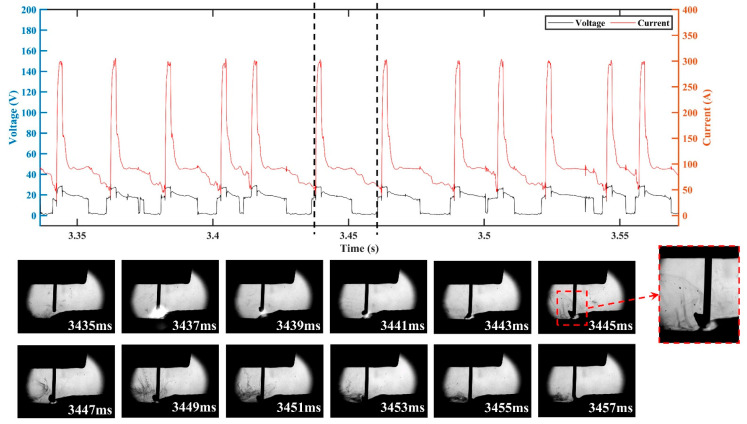
Electrical waveforms and droplet transfer process at I_sc_wait = 90 A.

The electrical signal waveforms and the corresponding droplet transfer process at I_sc_wait of 90 A (i.e., sample number 13) are shown in Figure 9. There were excessively long CMT cycles with abnormal short-circuit transition, leading to an increase in *IV_SC_*. The corresponding droplet transfer process showed that the droplet was spattered when it approached the molten pool, thus preventing the contact between the droplet and the molten pool. According to Equation (2), the excessive base current (i.e., high I_sc_wait) resulted in an increase in the arc force; thus, the repulsion of the droplet closer to the molten pool became more obvious, which not only resulted in a longer base phase but also caused spattering in an outward direction along the axis of the arc, thus destabilizing the CMT deposition process.

**Figure 10:**
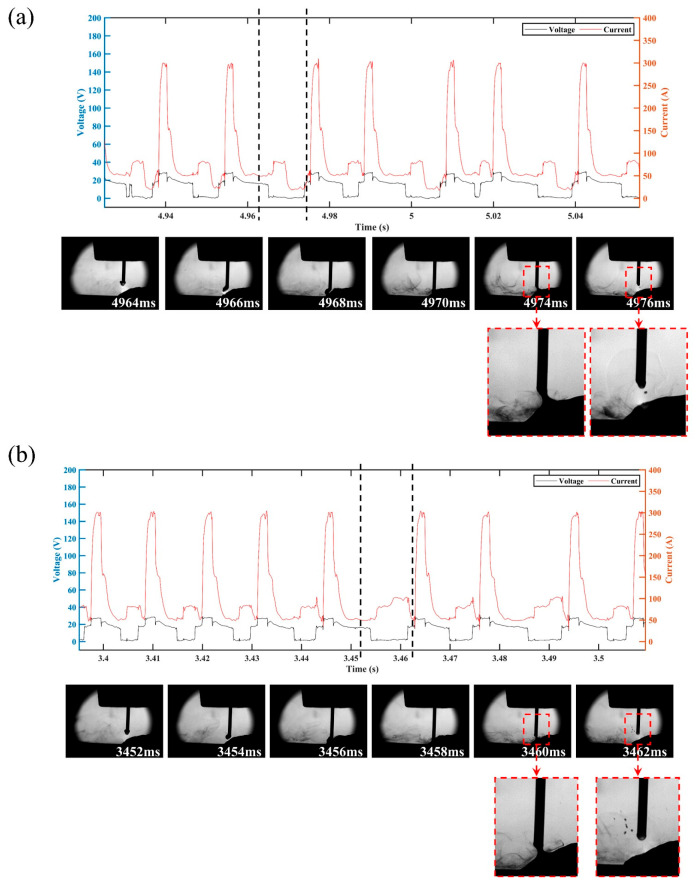
Electrical waveforms and droplet transfer process at (**a**) I_sc2 = 20 A and (**b**) I_sc2 = 100 A.

Figure 10 shows the electrical signal waveforms and the corresponding droplet transfer process at I_sc2 of 20 A (i.e., sample number 14) and I_sc2 of 100 A (i.e., sample number 17). Under the condition of low or high short-circuit current, the liquid bridge formed at the end of the short-circuit phase became thick, making the liquid bridge prone to spattering when it was pulled off. However, the inducements and spattering patterns were different under these two conditions. When the short-circuit current was low, the spatter was located in an area where the liquid bridge was pulled off, as shown in Figure 10a, due to the high viscosity coefficient of the liquid metal induced by the low heat input of the short-circuit phase. When the short-circuit current was high, a relatively high current was passed through the necking, which caused the liquid bridge to explode due to overheating and vaporize, resulting in spattering. Therefore, the spatter was repelled in the outward direction with respect to the liquid bridge axis, as shown in Figure 10b.

### 3.3. Optimization of the CMT Characteristic Parameters

The process stability of the CMT swing arc deposition of AZ91 magnesium alloy is affected by the four above-mentioned CMT characteristic parameters, so the selection of CMT characteristic parameters for process optimization must be implemented using a proven and reliable method. Design of experiment (DoE) can provide a scientific/statistical approach for evaluating process variables, and it has the advantage of possible reduction in the number of experiments required for establishing relationships between input and output parameters [36]. In this paper, an experiment based on the rotatable 3D central composite design was used to systematically establish the relationship between CMT characteristic parameters and the corresponding *IV_SC_*. For a set of measurable and controllable independent variables *x_k_*, the response variable *y* can be expressed as

(8) $${y=f\;(x}_{1}{,\;x}_{2}{,\;x}_{3},\;\ldots \ldots {\;,\;x}_{k})$$

Based on the previous analysis, large I_sc_wait increased the repulsion, so the instability caused by I_sc_wait could be avoided by keeping it at a small value, i.e., I_sc_wait of 10 A. Therefore, I_boost, t_I_boost and I_sc2 were selected as the input parameters, with *IV_SC_* as the output parameter. The range of the CMT characteristic parameters for the swing arc deposition process are presented in Table 4.

As aforementioned, the design of experiment used in this paper was based on a rotatable central composite design, and the number of experimental runs *n* can be determined with Equation (9):

(9) $${n=m}_{c}{\;+\;m}_{r}{\;+\;m}_{0}$$

where *m_c_* is the number of two-level trials (corner points) and its value can be determined with *m_c_ =* 2*^p^*; *m_r_* is the number of trials at the asterisk (center points) and its value can be determined with *m_r_* = 2*p*; *m*_0_ is the number of zero-level trials (original point) and its value can be determined with *m*_0_ *= r*^4^; *p* is the number of input factors; and *r* is the length of the asterisk arm. As mentioned, the number of input factors *p* was 3, while the length of the asterisk arm *r* was 1.682 in the three-factor central composite design. Therefore, *m_c_*, *m_r_* and *m*_0_ were 8, 6 and 9, respectively, and the number of experimental runs *n* was 23.

In this paper, the three experimental factors, i.e., I_boost, t_I_boost and I_sc2, were recorded as *x*_1_, *x*_2_ and *x*_3_, respectively, and *IV_SC_* was recorded as *y*. The upper and lower bounds of the *j*th factor are *Z_2j_* and *Z_1j_* (*j* = 1, 2, …, *p*), respectively, and the interval of zero-level variation $Z_{0j}$ and the variation spacing $\Delta_{j}$ can be calculated according to Equations (10) and (11):

(10) $$Z_{0j}=\frac{Z_{1j\;}{+Z}_{2j}}{2}$$

(11) $$\Delta_{j}=\frac{Z_{2j}-Z_{0j}}{r}=\frac{Z_{2j}-Z_{1j}}{2r}$$

The true values of the corresponding level codes of I_boost, t_I_boost and I_sc2 were calculated according to Equations (11) and (12) respectively, and the results are shown in Table 5. The design table of the CMT characteristic parameters and the corresponding *IV_SC_* are presented in Table 6. The CMT characteristic parameters for the swing arc deposition process were set based on a trial of a rotatable 3D central composite design.

In the case of *p* = 3, mathematical models based on second-order polynomials were developed for predicting the responses. The models can be expressed as follows:

(12) $${y=b}_{0}+\sum_{j=1}^{3}b_{j}x_{j}+\sum_{k=1}^{2}\sum_{j=2}^{3}b_{kj}x_{k}x_{j}+\sum_{j=1}^{3}b_{jj}x_{j}^{2}$$

where *x*_1_, *x*_2_ and *x*_3_ correspond to the three experimental input variables, i.e., I_boost, t_I_boost and I_sc2, respectively; *y* is the response variable, i.e., *IV_SC_*; and *b*_0_, *b_j_*, *b_k_* and *b_jj_* are the regression coefficients. Therefore, the mathematical model based on the results in Table 6 is as follows:

(13) $$y=0.39-0.068Z_{1}-0.066Z_{2}\;-0.014Z_{3}-0.049Z_{1}Z_{2}-0.0055Z_{1}Z_{3}\;\;\;\;\;\;\;\;\;\;\;\;\;\;\;-0.001Z_{2}Z_{3}-0.019Z_{1}^{2}+0.00446Z_{2}^{2}+0.006227Z_{3}^{2}$$

Significance tests are required to ensure the credibility and fit of the regression equation. The analysis of variance for *y* is presented in Table 7. *SS* and *df* are the sum of squares and the degrees of freedom, respectively, and *MS* is the mean square calculated with *SS*/*df*. *F* indicates the significance, and a larger *F* represents a more significant variable and a better fit of the regression. *P* is the probability used to test the hypothesis at the 95% confidence level in this paper, and when its value is less than 0.05, it indicates that the factor is significant.

The results in Table 7 show that the *F* of the model is 23.39, indicating that the model is significant. For the condition of *P* less than 0.05, *Z*_1_, *Z*_2_, *Z*_1_*Z*_2_ and *Z*_1_^2^ are the terms with significant impact. Therefore, by removing the insignificant terms, the optimized regression equation can be obtained as follows:

(14) $$y=0.39-0.068Z_{1}\;-0.066Z_{2}-0.049Z_{1}Z_{2}-0.019Z_{1}^{2}$$

The coefficient of determination *R*^2^, as the percentage of the variability, indicates the degree of prediction of the regression model, and its maximum value is 1. The *R*^2^ of Equation (14) is 0.8998, so the optimized regression equation has a high goodness of fit. By converting the above regression equation from the coded space to the real space, it can be expressed as follows:

(15) $$y=-0.4591+0.00690518x_{1}\;+0.42933x_{2}-0.0023007x_{1}x_{2\;}-0.00000860779x_{1}^{2}$$

**Figure 11:**
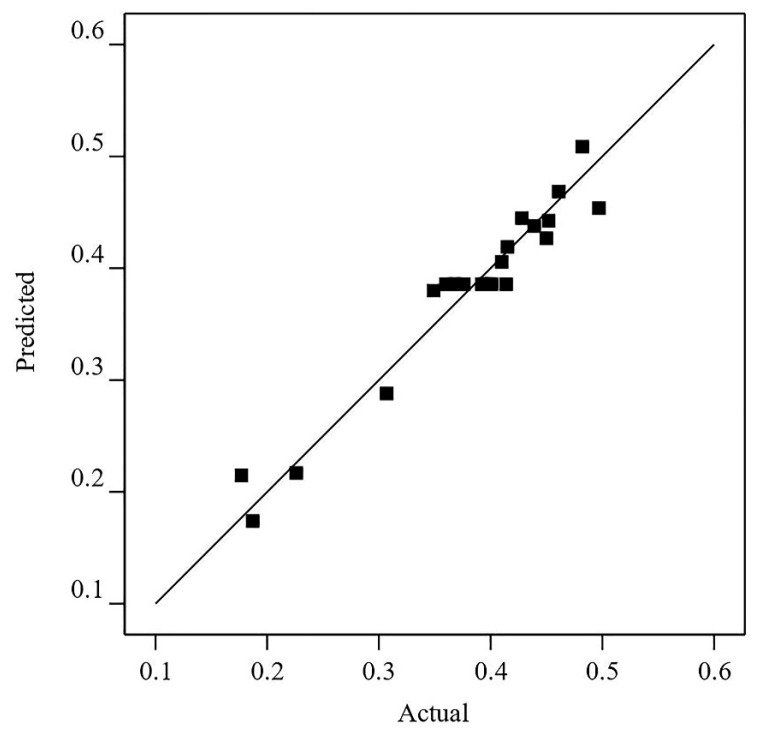
Predicted values versus actual values of *IV_SC_*.

The straight line in Figure 11 represents the predicted value of *y* (i.e., *IV_SC_*) equal to its actual values. As shown in Figure 11, the actual values of *y* for the tests in Table 6 are distributed around the straight line, which can also indicate that the obtained regression equation fits significantly. Therefore, it can reflect the relationship between the CMT characteristic parameters and *IV_SC_*.

**Figure 12:**
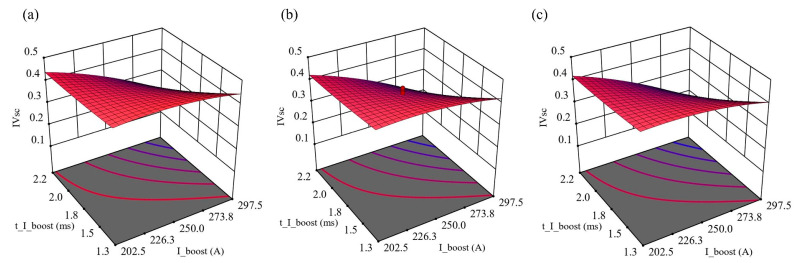
Response surface of I_ boost, t_ I_boost and *IV_SC_* at different I_sc2: (**a**) I_sc2 = 58 A; (**b**) I_sc2 = 70 A; (**c**) I_sc2 = 82 A.

As indicated by *IV_SC_* with different CMT characteristic parameters, a regression model was established to predict the process stability of swing arc deposition. The results in Table 7 show that the effect of I_sc2 on the model is insignificant. The response surface of I_ boost, t_ I_boost and *IV_SC_* at different I_sc2 levels within its range in Table 5 (i.e., 50–90 A) was established, and the results are shown in Figure 12. Using the response surface method, the experimental errors can be taken into account, and the complex functional relationships can be fitted to a continuous surface within a certain region. Thus, the optimal value of *IV_SC_* with the lowest value can be found.

**Figure 13:**
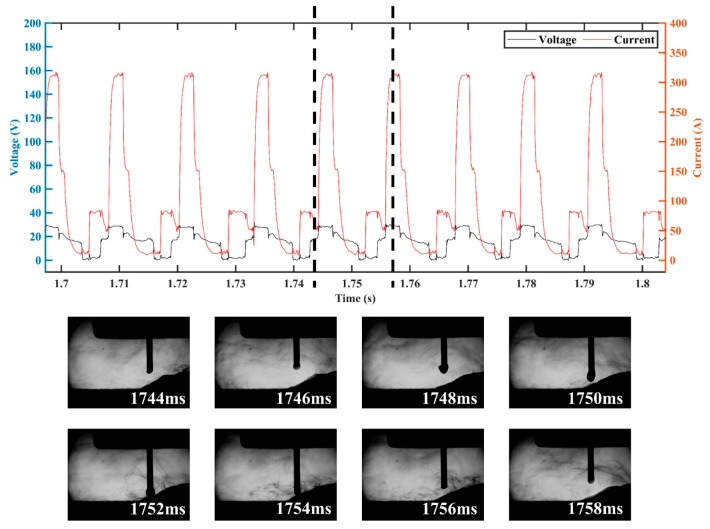
Electrical waveforms and droplet transfer process with the optimum CMT characteristic parameters.

Table 8 shows the optimal combination of the CMT characteristic parameters (i.e., I_ boost, t_ I_boost and I_sc2) obtained with the established prediction model, as well as the corresponding predicted and actual *IV_SC_*. Figure 13 shows the electrical signal waveforms and droplet transfer process of the deposited layer obtained using the optimum parameters in Table 8. As indicated in Figure 13, the electrical waveform was stable and free of abnormalities, and the phenomenon of droplet deviation during the CMT swing arc deposition process of Mg alloy could be eliminated using the optimized characteristic parameters.

**Figure 14:**
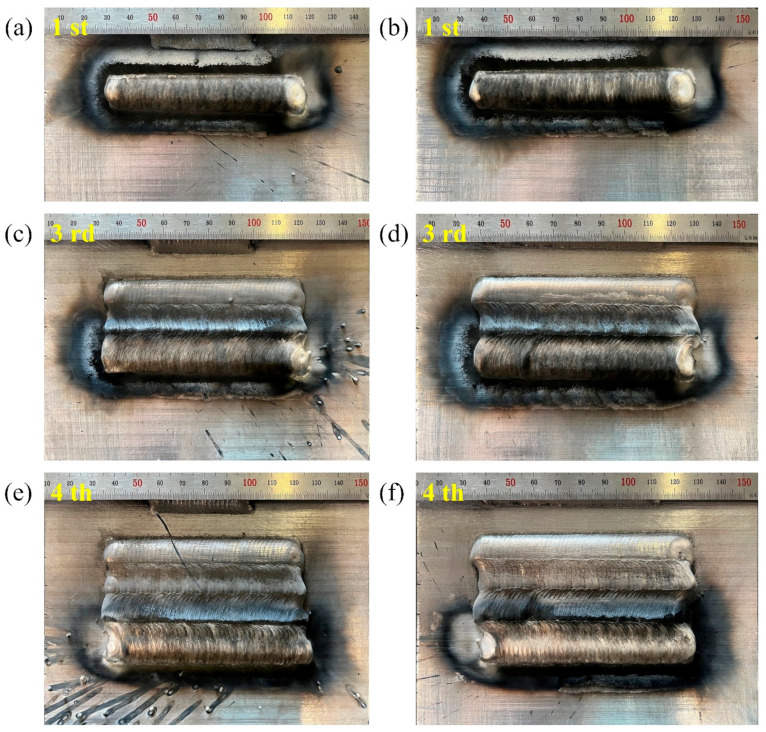
Appearance comparison: (**a**,**c**,**e**) 1, 3 and 4 deposited passes obtained before optimization, respectively; (**b**,**d**,**f**) 1, 3 and 4 deposited passes obtained after optimization, respectively.

**Figure 15:**
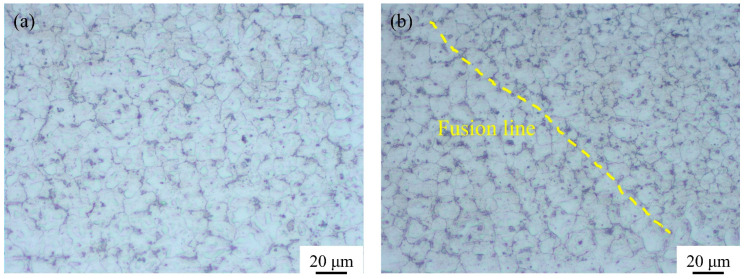
Microstructure of deposited layer: (**a**) interior of the deposited layer; (**b**) interface between successive deposited layers.

As shown in Figure 14, the generation of spatter could be effectively reduced during the multi-pass deposition process. Figure 15 shows that the deposited layer was composed of equiaxed grains, and there were significant differences in the average grain size in the transition zone between the adjacent deposited layers. The previous deposited layer could provide good heat dissipation conditions for the subsequent deposited layer, resulting in the formation of fine equiaxed grains on one side of the fusion line. Meanwhile, the previous deposited layer was reheated during the deposition process of the subsequent layer, leading to the formation of coarse equiaxed grains in the former layer near the fusion line. Furthermore, a defect-free microstructure was obtained, indicating good quality of the deposited layer and the interface of the successive deposited layers. In sum, a stable process of CMT swing arc deposition without spatter could be realized using the optimum parameters obtained with the established prediction model, and the deposited layers showed good appearance with no obvious defects.

## 4. Conclusions

In this paper, a stable deposition process was realized by optimizing the CMT characteristic parameters, i.e., I_sc_wait, I_boost, t_I_boost and I_sc2. The primary conclusions obtained in this study are as follows:

(1) During the swing arc deposition process, a horizontal component of the arc force was formed, thus significantly affecting the stability of the droplet transition.
(2) The Vilarinho regularity index for short-circuit transfer (*IV_SC_*) based on the coefficient of variation could be used to characterize the stability of the deposition process. I_sc_wait had a linear function relation with *IV_SC_*, while I_boost, t_I_boost and I_sc2 all presented a quadratic correlation with *IV_SC_*.
(3) A prediction model of *IV_SC_* was established based on a rotatable 3D central composite design method; then, the optimization of the CMT characteristic parameters could be realized using this model.
(4) The optimum combination of CMT characteristic parameters for the swing arc deposition process of Mg alloy was I_boost of 312.5 A, t_I_boost of 2.5 ms, I_sc_wait of 10 A and I_sc2 of 75.5 A, which could guarantee a stable deposition process and good appearance of the defect-free deposited layer.

## Figures and Tables

**Table 1:** Chemical composition of the substrate plate and wire (wt. %).

Material	Al	Zn	Mn	Si	Fe	Cu	Ni	Mg
Base metal plate	8.70	0.58	0.24	0.02	0.002	0.005	0.001	Bal.
Wire	8.62	0.55	0.33	0.0067	0.0040	0.00019	0.00023	Bal.

**Table 2:** CMT characteristic parameters for the swing arc deposition of Mg alloy.

Sample Number	I_boost (A)	t_I_boost (ms)	I_sc_wait (A)	I_sc2 (A)
1	300	2	50	60
2	100	2	50	60
3	150	2	50	60
4	400	2	50	60
5	450	2	50	60
6	300	0.5	50	60
7	300	1	50	60
8	300	3	50	60
9	300	4	50	60
10	300	2	10	60
11	300	2	30	60
12	300	2	80	60
13	300	2	90	60
14	300	2	50	20
15	300	2	50	40
16	300	2	50	80
17	300	2	50	100

**Table 3:** Phase duration, heat input and *IV_SC_* with different CMT characteristic parameters.

Sample Number	Boots Phase (ms)	Burn Phase (ms)	S/C Phase (ms)	CMT Cycle (ms)	Heat Input (J/mm)	*IV_SC_*
1	1.995	6.732	3.620	12.247	159.749	0.264
2	3.048	4.519	4.399	11.966	82.270	0.393
3	3.044	4.454	3.832	11.330	102.832	0.293
4	1.924	7.804	3.883	13.610	188.64	0.334
5	1.973	9.084	4.240	15.198	201.647	0.458
6	0.464	6.789	3.407	10.560	104.617	0.307
7	0.937	6.557	3.426	10.820	117.957	0.258
8	2.962	7.246	4.266	14.374	181.512	0.309
9	3.940	8.037	4.544	16.422	226.065	0.464
10	2.001	6.019	3.385	11.305	161.453	0.154
11	1.989	6.165	3.367	11.421	159.789	0.220
12	1.975	8.184	4.233	14.291	156.514	0.416
13	1.978	10.147	5.836	17.862	150.157	0.457
14	1.994	7.080	4.257	13.231	151.995	0.370
15	1.993	6.834	3.794	12.520	153.177	0.304
16	1.992	6.670	3.622	12.184	157.179	0.268
17	1.995	6.817	3.907	12.619	158.564	0.293

**Table 4:** Range of the CMT characteristic parameters.

Deposition Parameter	I_boost (A)	t_I_boost (ms)	I_sc2 (A)
Parameter range	170–330	1–2.5	50–90

**Table 5:** Variables and levels for the CMT characteristic parameters.

Level	I_boost (A)	t_I_boost (ms)	I_sc2 (A)
+1.682	330	2.5	90
+1	297.5	2.2	82
0	250	1.8	70
−1	202.5	1.3	58
−1.682	170	1	50

**Table 6:** Design table of the CMT characteristic parameters and the corresponding *IV_SC_*.

Sample Number	*Z*_1_	*Z*_2_	*Z*_3_	I_boost (A)	t_I_boost (ms)	I_sc2 (A)	*IV_SC_*
1	1	1	1	297.5	2.2	82	0.187
2	1	1	−1	297.5	2.2	58	0.177
3	1	−1	1	297.5	1.3	82	0.410
4	1	−1	−1	297.5	1.3	58	0.452
5	−1	1	1	202.5	2.2	82	0.415
6	−1	1	−1	202.5	2.2	58	0.439
7	−1	−1	1	202.5	1.3	82	0.497
8	−1	−1	−1	202.5	1.3	58	0.461
9	1.682	0	0	330	1.8	70	0.226
10	−1.682	0	0	170	1.8	70	0.428
11	0	1.682	0	250	2.5	70	0.307
12	0	−1.682	0	250	1	70	0.482
13	0	0	1.682	250	1.8	90	0.349
14	0	0	−1.682	250	1.8	50	0.450
15	0	0	0	250	1.8	70	0.401
16	0	0	0	250	1.8	70	0.376
17	0	0	0	250	1.8	70	0.395
18	0	0	0	250	1.8	70	0.414
19	0	0	0	250	1.8	70	0.367
20	0	0	0	250	1.8	70	0.360
21	0	0	0	250	1.8	70	0.392
22	0	0	0	250	1.8	70	0.369
23	0	0	0	250	1.8	70	0.399

**Table 7:** Analysis of variance for response *y*.

Source	*SS*	*df*	*MS*	*F*	*p*	
Model	0.15	9	0.017	23.39	<0.0001	Significant
*Z*_1_	0.063	1	0.063	87.60	<0.0001	Significant
*Z*_2_	0.059	1	0.059	82.12	<0.0001	Significant
*Z*_3_	0.002639	1	0.002639	3.68	0.0771	
*Z*_1_ *Z*_2_	0.019	1	0.019	27.09	0.0002	Significant
*Z*_1_ *Z*_3_	0.000242	1	0.000242	0.34	0.5710	
*Z*_2_ *Z*_3_	0.000008	1	0.000008	0.011	0.9175	
*Z*_1_^2^	0.005983	1	0.005983	8.35	0.0126	Significant
*Z*_2_^2^	0.000316	1	0.000316	0.44	0.5182	
*Z*_3_^2^	0.0006162	1	0.0006162	0.86	0.3706	

**Table 8:** Optimization of the CMT characteristic parameters and the corresponding *IV_SC_*.

Characteristic Parameters	I_boost (A)	t_I_boost (ms)	I_sc2 (A)	Predicted *IV_SC_*	Actual *IV_SC_*
Optimum	312.5	2.5	75.5	0.134	0.153

## Data Availability

The raw/processed data required to reproduce these findings cannot be shared at this time due to technical or time limitations.
